# Secondary Traumatic Stress in Foster Carers: Risk Factors and Implications for Intervention

**DOI:** 10.1007/s10826-019-01668-2

**Published:** 2019-11-28

**Authors:** Kay M. Bridger, Jens F. Binder, Blerina Kellezi

**Affiliations:** Nottingham Trent University, Nottingham, UK

**Keywords:** Foster carers, Looked after children, Secondary trauma, Indirect trauma, Self-care

## Abstract

**Objectives:**

Fostering, a professional or semi-professional role that is in increasing demand, involves potential exposure to material related to children’s trauma in a domestic setting. Yet, professional vulnerability to secondary traumatic stress (STS) is under-researched in foster carers, as is the suitability of associated intervention techniques. We therefore investigated incidence of STS and psychological predictors relevant to secondary and primary stress appraisal in UK foster carers.

**Methods:**

British foster carers (*n* = 187; 81% female; aged 23–72 years; mean length of experience 9 years) were approached through a range of organizations managing paid foster caring in the UK for a survey study. Self-report measures were obtained on STS, burnout and compassion satisfaction from the Professional Quality of Life (ProQOL) scale, as well as on primary trauma and variables previously recommended for inclusion in training targeting secondary trauma: empathy, resilience and self-care.

**Results:**

High levels of STS and burnout were found among foster carers. In multivariate model testing, STS was directly and positively predicted by burnout, compassion satisfaction and primary trauma (*R*^2^ = 0.54, *p* < 0.001). Resilience, empathy and self-care did not show direct associations with STS, but self-care had a significant indirect effect on STS.

**Conclusions:**

Findings support the view that STS is a substantial risk factor in foster caring. While self-care is confirmed as a promising factor in intervention, the roles of empathy and resilience are more ambiguous.

In the US an estimated 437,465 children were in foster care in 2016, 45% of whom were in non-relative foster family homes (Child Welfare Information Gateway 2018). The number of children in care in England alone in 2017 was 72,670, of whom the majority (around 74%) were placed in foster care (Children’s Commissioner for England 2018). The need for foster carers, whose role it is to provide a nurturing home of varying duration to children in public care, is increasing in the UK (Department for Education 2017) but worryingly 12% leave the role annually (The Fostering Network 2016). In terms of primary trauma, 48% of UK foster carers reported physical harm from a foster child (Hannah and Woolgar 2018). Other evidence highlights the importance of secondary trauma (Figley 1995) as foster carers are exposed to the trauma histories of vulnerable children and young people. In the UK, 62% of fostered children in 2016/17 had prior experience of neglect or abuse (Department for Education 2017) facilitating trauma histories. Sustained exposure to primary and secondary trauma, the latter being much less understood, might reduce carer capacity to engage with foster children and retention of foster carers.

The impact of secondary trauma through exposure to client trauma in helping professionals has been evidenced with psychotherapists working with sexual abuse survivors (McCann and Pearlman 1990), social workers (Michalopoulos and Aparicio 2012), and child-focused professions such as child welfare workers (Sprang et al. 2011). Although of mounting importance, comparatively little attention has been given to the risk and protective factors surrounding secondary trauma among foster carers, and the trauma training currently targeted at foster carers (AC Education 2018; Simply Fostering Consultancy 2018). Gathering empirical support for a multivariate model of secondary traumatic stress (including a range of antecedent variables suggested by the STS literature), specifically obtained in the context of foster carers, is vital for informing further preventative training and support to this group.

The available research on the impact of secondary, or indirect, trauma and related concepts lacks clarity (Kadambi and Ennis 2008; Knight 2013) with several overlapping constructs, including secondary traumatic stress (STS; Stamm 2010), compassion fatigue (CF; Figley 1995) and vicarious trauma (VT; McCann and Pearlman 1990), used interchangeably or even in combination across the literature. STS has been defined as stress responses including PTSD-like symptoms in response to client trauma material (Figley 1995). Underpinning theory defined CF as synonymous with STS, but CF is frequently measured as a combination of STS and burnout and is seen as an absence of the positive aspects of professionals’ experience, such as the pleasure derived from helping others (Stamm 2010). Burnout in turn is defined as work-related exhaustion (Maslach et al. 2001), but without trauma-specific causation. VT has been defined as a more chronic disruption of cognitive beliefs, with a focus on changes in the therapist’s enduring ways of experiencing self and others (McCann and Pearlman 1990).

Lazarus and Folkman’s (1984) Model of Stress and Coping outlines processes of primary and secondary stressor appraisal. We assume that the potential threat of stressors (such as secondary trauma) is appraised relative to personal and environmental coping resources and that successful or unsuccessful coping leads to ongoing reappraisal of stressors. Burnout, STS and CF all indicate negative ability to cope with stressors. Minnis and Devine (2001) found foster carers disturbed by abuse disclosures (constituting secondary trauma exposure), with those with 30 or more child placements being the most likely to experience placement breakdown. They speculated that this may be influenced by foster carer burnout. Ottaway and Selwyn (2016) evidenced above average risk of compassion fatigue (CF) as well as burnout in a sample of 546 foster carers, while Hannah and Woolgar (2018) evidenced high rates of both STS and burnout among UK foster carers.

Regarding the relationship between burnout and STS, a meta-analysis of professionals working with trauma survivors (Cieslak et al. 2014) found strong associations between burnout and STS, suggesting this may be due to both constructs sharing the same risk factors. Longitudinal studies have found a unidirectional relationship, with earlier burnout predicting increased risk of later STS but not vice-versa (Kotaro et al. 2015; Shoji et al. 2015). Thus, burnout may deplete resources which in turn mitigate STS development, an explanation congruent with Lazarus and Folkman’s (1984) theory of stressor appraisal being influenced by, and influencing, coping resources.

Among the immediate correlates of STS, then, the CF construct seems to add comparatively little, both on a conceptual and an empirical level, over and above STS and burnout. STS and burnout have been separated at the level of theory and measurement by explicit inclusion or exclusion of trauma-related symptoms. Moving forward, CF should be omitted when the focus is on investigating STS and burnout due to the risk of creating conceptual redundancy. Closely related to CF, however, is compassion satisfaction, which has been discussed in past work as a protective factor.

Responses to indirect trauma are not always defined by negative outcomes, as the stressor appraisal and its impact is grounded in the availability of coping resources and subjective primary appraisals of distress (Lazarus and Folkman 1984). Compassion satisfaction (CS) is a possible coping resource which may ameliorate exposure to indirect trauma, as are the following three factors previously found to negatively predict STS in professions working with trauma populations: resilience among oncology nurses (Potter et al. 2013), self-care among residential childcare workers (Eastwood and Ecklund 2008) and empathy among social workers (Wagaman et al. 2015).

Various accounts for the relationship between Compassion Satisfaction (CS) and STS have been proposed. The CS construct reflects positive professional experiences (primary appraisal), mitigating negative experiences that lead to STS (Hinderer et al. 2014; Lee et al. 2015; Stamm 2010) or as a positive pathway to STS resilience (Ludick and Figley 2017), and both pathways could be relevant simultaneously.

Psychological resilience has been described as the capacity to bounce back from life stressors (Kapoulitsas and Corcoran 2015; Ong et al. 2006), either as a heritable personality trait or the product of other factors such as experience of positive emotions. The latter conceptualisation has similarities to Stamm’s (2010) description of CS and might be cultivated through training. Harker et al. (2016) found that STS scores in human service professionals declined following an intervention to promote resilience and mindfulness, while Kapoulitsas and Corcoran’s (2015) qualitative research with social workers indicated that the relationship between STS and resilience was more complicated and context-dependent, including the role of empathy.

Canfield (2005) asserted that self-care could prevent exposure to STS from developing into a chronic disorder. In support, Itzhaki et al. (2015) found that self-care moderated burnout and CF in nurses in a five-country study. This could be interpreted relative to Kotaro et al. (2015) and Lazarus and Folkman’s (1984) theories that burnout depletes resources; self-care may build up (coping) resources which reduce the likelihood of exposure to traumatic material resulting in STS. Ludick and Figley (2017) included self-care as a predictor of CF resilience. Eastwood and Ecklund (2008) found that only specific self-care practices ameliorated CF in childcare workers.

Foster caring is founded on interpersonal relationships and so it is plausible to assume a general relevance of empathy. Empathy is the ability to understand other peoples’ emotional experience (Myszkowski et al. 2017). Figley (1995) proposed empathy as a key cause of helping professionals’ vulnerability to STS, but evidence is mixed. Research has shown empathic perspective-taking to be protective against CF and burnout in nurses (Yu et al. 2016) and empathy to moderate STS in trauma workers (MacRitchie and Leibowitz 2010). Only some, and different, aspects of empathy predicted STS, CS and burnout among social workers (Wagaman et al. 2015). Turgoose et al. (2017) failed to establish a link between empathy, STS and CF among police officers working with rape victims. Furthermore, studies have pointed to the paradoxical effect of empathy acting as both risk and protective factor (Ludick and Figley 2017).

The STS construct focuses only on traumatic stress from indirect exposure to trauma, but it might be possible that threat of harm, or actual harm, to self or a member of the household while fostering could also result in PTSD-like symptoms and STS. Exposure to violence has been found to correlate positively with STS and PTSD scores in psychiatric nurses (Zerach and Shalev 2015) suggesting that delineation of primary and secondary trauma may not be clear-cut. A recent meta-analysis of STS in therapeutic professionals (Hensel et al. 2015) concluded that consistent associations with STS were present for occupational exposure to trauma material as well as personal history of trauma. Further, it needs to be recognised that the specific context of foster caring can lead to unique contextual pressures that lead to potentially different outcomes in comparison to other helping professions. In particular, the separation of emotional and professional life as a way of controlling exposure to trauma material would seem unlikely among foster carers.

The present study seeks to test the model shown in Fig. 1. Our model investigates the contribution (direct and indirect) of a range of predictors to secondary traumatic stress outcomes in foster carers. STS is treated here as the immediate trauma-related outcome, separated from burnout. Burnout and compassion satisfaction are included as contextual predictors of STS. Predictors of empathy, resilience and self-care which have been applied in STS-focused training interventions with other helping professionals are included as prospective coping resources. Incidence of primary trauma is included as an additional predictor with the potential to impact STS in this population. Greater understanding of the associations between these predictors will benefit local authorities and independent agencies which manage foster carers as they seek to promote role retention by reducing the negative impacts of fostering and promoting positive aspects such as compassion satisfaction.

**Fig. 1:**
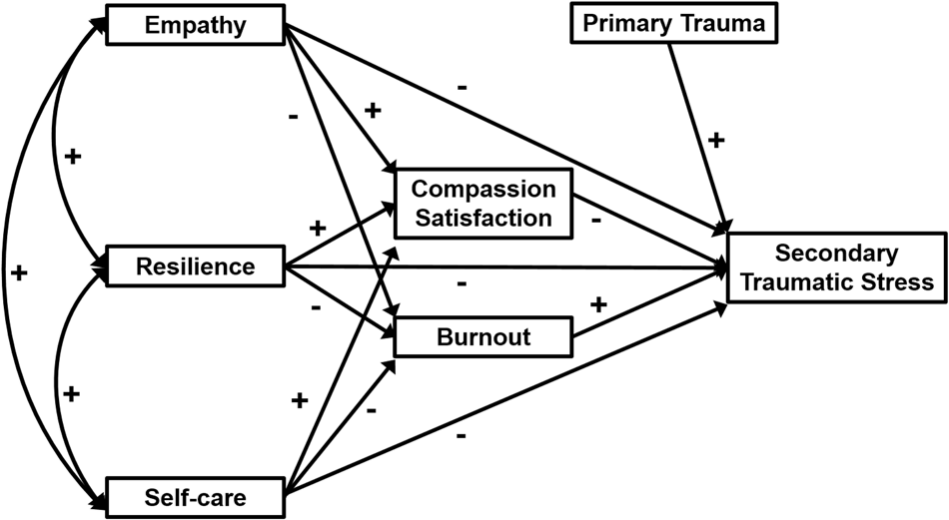
Prospective model of direct and indirect predictors of secondary traumatic stress in foster carers. Curved double-headed arrows represent covariation among variables

## Method

### Participants

Across the UK, 187 individuals currently working as foster carers at their private homes or in residential homes for young people in care took part in the survey. Nine organisations managing paid carers of children in public care distributed the survey link to relevant individuals: three local authorities, two independent fostering agencies, three charities or not-for-profit agencies and one residential care home. Seven further organisations were approached but did not respond. Although most residential workers do not normally live on the premises, their role still involves engaging with children over time, sharing their daily lives and being potentially exposed to children’s trauma. They were therefore included in the present study. Demographic information for the sample is presented in Table 1. Disclosed employers were based almost exclusively in England, with four stating they fostered in Wales or Scotland. The sample were predominately female (*n* = 152, 81%), with a mean age of 50 years and an average foster carer experience of 9 years. The sample had similar demographics compared to Hannah and Woolgar (2018) and Ottaway and Selwyn (2016) and is very close to the known demographics of the current foster carer population in the UK (The Fostering Network 2019).

**Table 1:** Demographic characteristics of the sample

Sample (*n* = 187)	*n*	%
Sex		
Female	152	81.3
Male	33	17.6
Undisclosed	2	1.1
Age in years (*M* = 50.37, *SD* = 9.10)		
23–39	23	12.3
40–49	58	31.0
50–59	79	42.3
60–72	27	14.4
Fostering employer		
Local authority	91	48.7
Charitable organization	35	18.7
Independent agency	33	17.6
Did not identify	28	15.0
Fostering experience (*M* = 8.57, *SD* = 7.12, Range [2 weeks, 33 years])		
<2 years	27	14.4
2–5 years	55	29.4
6–10 years	45	24.1
11–20 years	47	25.1
>20 years	12	6.4
Undisclosed	1	0.5
Regrets decision to foster		
Yes	27	14.4
Maybe	37	19.8
No	121	64.7
Undisclosed	2	1.1

### Procedure

A cross-sectional survey measured STS as the primary outcome variable and burnout, CS, empathy, resilience, self-care as well as primary trauma as wider explanatory variables. Demographic items included age, sex, fostering employer and length of fostering experience. The survey was piloted and discussed with three current foster carers before distribution. Foster carers were surveyed via an online survey platform (Qualtrics). Respondents were informed of the inclusion of potentially distressing questions at the start of the survey, and they were directed to support resources specific to foster carers in the UK.

### Measures

The following variables were measured based on validated questionnaire scales.

#### Secondary traumatic stress, burnout and compassion satisfaction

Three elements of professional experience were measured with the widely used and validated ProQOL-V instrument (Stamm 2010), a 30-item self-report questionnaire with three subscales and good internal consistency (Hannah and Woolgar 2018). The ProQOL asks respondents to consider the frequency of their experiences in their work situation over the past 30 days, rated from 1 (*never*) to 5 (*very often*) with higher average values indicating higher STS, burnout and CS. The ProQOL is used to identify risk and is not considered diagnostic (Stamm 2010). Ten STS items (α = 0.82) focused on PTSD-like symptoms consistent with the DSM-V (American Psychiatric Association 2013) such as hypervigilance, negative mood, avoidance and intrusion (e.g., “I avoid certain activities or situations because they remind me of frightening experiences of the people I help”). Ten items assessed burnout (α = 0.79) predominantly as affect ratings related to wellbeing (e.g., “I am happy.”) and to the work situation (e.g., “I feel trapped by my job as a foster carer.”), including aspects of work overload and attitudes towards the work role. Ten items assessed CS (α = 0.88) as the quantified professional pleasure and experienced benefit derived from helping others (e.g., “I feel invigorated after working with those I help.”).

#### Empathy

The Toronto Empathy Questionnaire (TEQ; Spreng et al. 2009) is a 16-item self-report unidimensional measure reflecting a range of empathy-related behaviours and skills. Items address facets of empathy including emotion comprehension in others, sympathetic physiological arousal, and altruistic behaviour on a scale from 0 (never) to 4 (always) with higher average values indicating higher empathy. The TEQ has previously shown good levels of internal consistency (Gould and Gautreau 2014; Spreng et al. 2009; α = 0.74 for the present study).

#### Resilience

The CD-RISC-10 (Campbell-Sills and Stein 2007), a 10-item scale (α = 0.85), assesses capacity to tolerate and adapt to challenging life experiences including change, personal problems, pressure, failings and painful feelings (e.g., “I tend to bounce back after illness or hardship.”). Statements are scored from 0 (almost never true) to 4 (almost always true) with higher average values indicating higher levels of resilience.

#### Self-care

Fifteen items with a clear focus on the psychological facet of self-care were taken from two existing scales. Eleven items from Dorociak et al.’s (2017) Professional Self-Care Scale were included: four life balance items referring to time spent socially with friends and family, four cognitive strategies items relating to mindful awareness of internal states including stress, and three daily balance items referring to deliberately making time for non-work activities. Four questions from the Trauma-Informed Self-Care (TISC) measure by Salloum et al. (2015) were included to capture the aspect of (semi-)professional support relevant to work with children. Three of the TISC measure items related to accessing supervision-like consultation, peer support, and training on secondary trauma. One question asked about use of meditation or mindfulness for stress management. All 15 items (α = 0.87) were answered on a seven point Likert scale (1 = *never;* 7 = *almost always*) with higher average values indicating higher self-care.

#### Incidence of primary trauma

In order to obtain a control measure for primary trauma, the Trauma History Screen (Carlson et al. 2011) was adapted to screen for potentially traumatic experiences during fostering. Experiences were defined as those with the potential to result in PTSD, i.e., witnessing harm to self or others, in line with the DSM-V (American Psychiatric Association 2013). Five Yes/No questions recorded incidence of deliberate harm to self or other by a member of the household, resulting in substantial injury of self or others, or death. Positive responses were summed up, yielding an index with a maximum score of 5, with any score greater than zero indicating exposure to primary traumatic incidents.

#### Exploratory questions

Next to the scale-based measures, two open-ended questions were asked “What most helps you to maintain your own wellbeing?” and “What support do you most need as a Foster Carer that is not available?”. Responses were used to derive more general themes, and themes were further merged in an iterative procedure to arrive at an informative number.

### Data Analyses

All quantitative analyses were carried out using SPSS v24 and AMOS v24. T-tests were conducted to investigate differences between female and male respondents. Bivariate product–moment correlations were computed for further inspection of associations among the study variables. Path analysis was used for multivariate model testing and to examine the direct and indirect contribution of predictors to secondary traumatic stress. Responses to two qualitative questions were coded through thematic analysis, following Braun and Clarke (2006).

## Results

Descriptive statistics for all study variables are presented in Table 2. In order to test for any gender differences, t-tests for independent samples were conducted on all variables. Only empathy showed a clear gender difference, with female respondents (*M* = 3.19, *SD* = 0.32) reporting higher levels of empathy than male respondents (*M* = 3.00, *SD* = 0.37, *t*(183) = 3.04, *p* < 0.01). In addition, STS showed differences approaching significance, with females (*M* = 2.54, *SD* = 0.62) reporting higher levels of traumatic stress than males (*M* = 2.31, *SD* = 0.51, *t*(183) = 1.97, *p* = 0.05).

**Table 2:** Descriptive statistics and bivariate correlations between study variables

	Age (1)	Experience (2)	STS (3)	Burnout (4)	CS (5)	Empathy (6)	Resilience (7)	Self-care (8)	PT (9)
Mean	50.4 (9.1)	8.57 (7.12)	2.50 (0.60)	2.36 (0.58)	4.11 (0.53)	3.16 (0.34)	2.98 (0.50)	4.31 (0.93)	1.82 (1.43)
Range		<1–33 years	1.20–4.20	1.00–4.40	2.40–5.00	2.19–3.88	1.60–4.00	1.87–6.67	0–5
(1)	–	0.41**	−0.10	−0.07	0.04	−0.13	−0.02	0.04	0.04
(2)		–	0.12	0.04	0.07	−0.04	0.00	0.02	0.38**
(3)			–	0.67**	−0.33**	−0.12	−0.23*	−0.28**	0.37**
(4)				–	−0.68**	−0.30**	−0.35**	−0.53**	0.25*
(5)					–	0.43**	0.40**	0.48**	−0.07
(6)						–	0.33**	0.23*	0.03
(7)							–	0.30**	0.14
(8)								–	−0.04
(9)									–

Standard deviations in parentheses next to mean scores

*p < 0.05, **p < 0.01

Bivariate correlations were inspected for all variables used in model testing as well as age and fostering experience (see Table 2). In terms of background characteristics, age only correlated with length of experience (*r* = 0.41, *p* < 0.001). Length of experience, in turn, was positively related to incidents of primary traumatic events (*r* = 0.38, *p* < 0.001). No other significant associations were found involving age or experience. The strongest associations overall were found for the ProQOL variables: between STS and burnout (*r* = 0.67, *p* < 0.001) and between burnout and CS (*r* = −0.68, *p* < 0.001). No indication of multicollinearity among the variables were suggested by the bivariate correlations.

### Professional Risk Levels in Foster Carers

In order to investigate levels of vulnerability in the sample, the cut-off scores for the top 25 percentiles were obtained for STS and burnout, along with the cut-off score for the lowest 25 percentiles for CS following the ProQOL manual (Stamm 2010). Cut-off scores were then compared to Stamm’s (2010) results obtained on 1187 caring professionals, used here as a reference sample. Sum scores were used, in line with the reference values, for this comparison. The cut-off for high levels of STS was higher among foster carers (29) than in the reference sample (17). Likewise, the cut-off for burnout was higher among foster carers (28) than in the reference sample (25). In other words, foster carers falling into the top 25 percent of the distribution showed higher reported STS and burnout when compared to the general sample described in Stamm (2010). The cut-off for CS, however, was higher among foster carers (37) when compared to the reference sample (32), suggesting overall increased compassion satisfaction, even among those foster carers who fell into the lower parts of the distribution.

### Multivariate Model Testing: Predicting STS

To provide a test for our prospective model predicting STS in foster carers, a path analysis was conducted using AMOS 24, modelling the structure outlined in Fig. 1. STS was predicted from burnout, CS, empathy, resilience, self-care and primary trauma. Indirect paths were included from empathy, resilience and self-care to STS via both burnout and CS. Empathy, resilience and self-care were allowed to co-vary, an assumption further supported by the correlations reported previously.

**Fig. 2:**
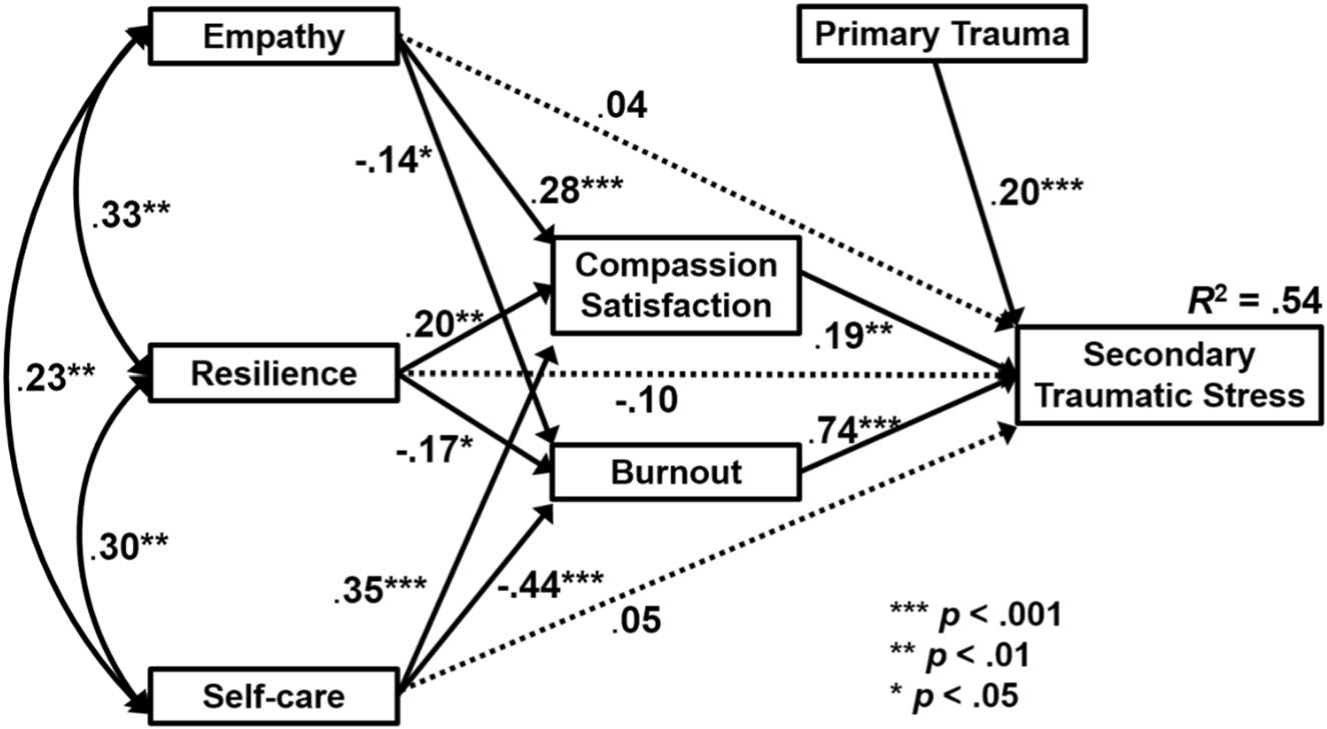
Empirical model of direct and indirect predictors of secondary traumatic stress in foster carers, obtained through structural equation modelling. Standardised coefficients are displayed. Solid arrows represent significant, dashed arrows non-significant paths

Results are shown in Fig. 2. Overall, the model predicted 54% of the variance in STS. In terms of direct effects, STS was most strongly associated with burnout (β = 0.74, *p* < 0.001), followed by primary trauma (β = 0.20, *p* < 0.001) and CS (β = 0.19, *p* < 0.01). In contrast to our expectations, CS positively predicted STS. This result also presented a change from the bivariate correlation between STS and CS, which had been found to be negative (see Table 2). All other associations in the model were in the expected direction, except for the direct paths from empathy, resilience and self-care to STS, which were all three not significant. Given that all paths from empathy, resilience and self-care to CS and burnout were significant, mediation effects were put to further tests. For this, indirect effects were estimated using bootstrapping in AMOS with 1000 iterations and bias-corrected 95% confidence intervals. A significant indirect effect was found for self-care on STS (95% CI [−0.34, −0.14], *p* = 0.04), suggesting that CS and burnout mediate a negative effect of self-care on STS. Neither for resilience (95% CI [−0.20, 0.01], *p* = 0.10) nor empathy (95% CI [−0.16, 0.05], *p* = 0.35) were indirect effects significant.

In sum, results confirmed model assumptions that CS and burnout were more proximate predictors of STS compared to empathy, resilience and self-care. While empathy, resilience and self-care all predicted CS and burnout as expected, they did not show any direct association with STS, and only for self-care was an indirect effect found.

### Reported Resources and Support Needs

Incidents of themes derived from the open-ended questions are displayed in Table 3. The response format in the study encouraged short answers, typically taking the form of part sentences or keyword-type entries. This necessarily meant that thematic complexity in the analyses was somewhat restricted. In response to the question “What most helps you to maintain your own wellbeing?” time was a predominant aspect. Time with others was most frequently mentioned (*f* = 58; e.g. “*my husband and older children*” “*friends and family*”) and the closely related theme of social support was likewise among the top 3 themes (*f* = 27; e.g. “*supportive close friends*”). This is complemented by the themes of time away (*f* = 18; e.g. “*time away from the foster child to relax*”) and time alone (*f* = 9; e.g. “*time for myself*”). In response to the question “What support do you most need as a Foster Carer that is not available?” it is important to note that 28 respondents stated no concerns. Time, again, was the most prominent theme named (*f* = 28; e.g. “*regular breaks/guilt free respite*”). Other themes clearly indicate a perceived lack of contributions from the (professional) environment: general professional support, specific training, and therapeutic support came up collectively 44 times (e.g. “*the offer of regular counselling/therapy if needed*”).

**Table 3:** Incidence of themes in answer to qualitative questions on wellbeing and support

What most helps you to maintain your own wellbeing?		What support do you most need as a Foster Carer that is not available?	
Time with others	58^a^	No concerns	28
Exercise	31	Time off/respite	28
Support	27	Professional support	25
Attitude/personality	25	Social worker issues	15
Time away	18	Other	13
Hobby/activities	17	Professional respect	10
Faith	13	Therapeutic	10
Rest	12	Specific training	9
Time alone	9	Financial support	6
Pamper/treats	6	Peer support	6
Reading	5	Case history	4
Training	4		
Mindfulness/meditation	4		
Diet	2		
Medication	1		

Frequency counts are reported

^a^Family was specifically mentioned as part of time with others 47 out of 58 times

## Discussion

In this study, we set out to investigate STS and its multivariate associations in a sample of foster carers. Our main findings can be summarised as follows. When compared to caregiver data provided by Stamm (2010) and to a previous foster carer sample (Hannah and Woolgar 2018), the present sample showed higher levels of secondary traumatic stress, burnout and CS. In addition, incidence of primary trauma was reported by 76.5% of respondents, a markedly higher rate compared to the 48% reported by Hannah and Woolgar’s (2018) regarding foster carers with experience of physical harm from a foster child, although the present study included more possible indicators of primary trauma. Regarding predictors of STS, our model explained 54% of the variance through burnout, primary trauma and CS. The prediction that empathy, resilience and self-care would contribute to STS variance directly was not supported. However, the three variables did significantly predict compassion satisfaction (all positively) and burnout (all negatively). Only self-care exerted a significant indirect effect on STS. Empathy, resilience and self-care were also positively correlated with each other and covarying in the model, suggesting some further interactions beyond the interpretation of the present model-testing study.

The finding that foster carers are at risk of secondary traumatic stress and burnout supports the need for further exploration of risk and preventative factors to inform support and training measures. The ProQOL measure is not diagnostic (Stamm 2010), but offers an interpretation of professional quality of life based on the relative scores of STS, burnout and compassion satisfaction. High STS coupled with high burnout is considered to indicate distress and a need for a break from the work environment when compassion satisfaction is low. The qualitative data brought up a frequent theme of an unmet need for “time off and/or respite from care”, in support of the reported levels of burnout. Compassion satisfaction, however, was higher than Stamm’s (2010) average, which may help to mitigate overall distress levels among the respondents.

Increased risk of secondary traumatic stress and burnout for foster carers is of wider concern to the public, as it may reduce role effectiveness and undermine retention. Given the predominantly female sample, it is notable that STS scores of females were marginally higher than those of males (see also Bride et al. 2009). The present sample’s gender balance was consistent with previous foster carer samples and may be a true reflection of a gendered workforce. If so, then sex differences in STS vulnerability may be of high relevance, as would be an under-representation of male foster carers. This could also reflect the wider social context whereby female vulnerability is strongly linked to gender role and gender-related expectations, in this case as carers, which affects both primary and secondary appraisal (Kellezi and Reicher 2014).

Regarding predictors of STS, our model provides a test of the contribution of burnout, CS, empathy, resilience, self-care and primary trauma. Burnout was expected to positively predict STS and CS to negatively predict it. In this sample, the majority of the STS variance was predicted by burnout, consistent with previous work (Devilly et al. 2009). Devilly et al. (2009) criticised the STS construct for possibly being conflated with burnout. While the highest bivariate correlations in the sample were between burnout and STS, multicollinearity was not indicated. The two ProQOL subscales were well differentiated, with burnout questions being unrelated to trauma material exposure. The correlation may instead be consistent with burnout and STS having similar risk factors (Cieslak et al. 2014), or with burnout contributing to resource depletion and thus increased vulnerability to STS, as suggested by longitudinal studies (Kotaro et al. 2015; Shoji et al. 2015) and, more generally, the Lazarus and Folkman (1984) stress model.

Given self-care, empathy and resilience all negatively predicted burnout in the path analysis, these three predictors could constitute resources which when depleted may no longer offer mitigation of STS development. The resource depletion theory offered by Kotaro et al. (2015) is taken as the most plausible explanation for the current findings, and depletion in turn contributes to the appraisal of secondary exposure to stressors, per Lazarus and Folkman (1984). That the same three predictors (self-care, empathy and resilience) positively contributed to CS in path analysis suggests that their overall contribution is more complex, however. Longitudinal evidence in foster carers would be required to draw further conclusions regarding causal directions of influence.

The positive contribution of CS to STS contradicted the proposed model and diverges from theory which suggests that CS mitigates STS (Stamm 2010; Hinderer et al. 2014). At the same time, and consistent with Hannah and Woolgar (2018), both STS and burnout scores were negatively associated with CS as far as bivariate correlations go. The positive contribution of CS to STS is not without precedent. Lee et al. (2015) found that higher CS predicted compassion fatigue in genetic counsellors. Their study notes that high CS and compassion fatigue can co-exist within individuals according to the ProQOL model (Stamm 2010) and suggests that individuals might use self-care strategies which build CS but do not reduce fatigue. In the complex foster carer role, it can be speculated that higher CS indicates greater role engagement which may result in greater exposure to trauma material and thus STS risk. This is supported in the present analysis by empathy’s strongest contribution being to CS. Empathetic engagement is a core element of Figley’s (1995) original theory of STS and the present data may indicate that it has both positive and negative impacts on professional quality of life. Foster carers may find that satisfaction from helping others through their work (primary appraisal) mitigates the development of burnout and STS, while empathetic engagement (as a coping strategy with side effects) simultaneously makes them more vulnerable.

Finally, the second largest predictor of STS variance was incidence of primary trauma. While primary trauma was high for the sample, the cross-sectional approach cannot confirm causal relationships between secondary and primary trauma indicators. Primary trauma was positively correlated with years of fostering, suggesting it to be a product of cumulative exposure. Experience has been associated with CF and STS in nurses (Yu et al. 2016), indicating some conflation between STS and primary trauma measurement. The ProQOL STS subscale claims to exclusively measure helping professionals’ PTSD-like symptoms resulting from exposure to client trauma (Stamm 2010), but in the present study it is difficult to differentiate between the PTSD-like symptoms of STS (Figley 1995) and PTSD symptoms from primary trauma. Research with helping professionals rarely measures exposure to primary trauma (Zerach and Shalev 2015) and so it has been little accounted for in the secondary trauma literature. If role-related primary trauma can be confirmed as a predictor of STS in future work, it would highlight a need to delineate measures of STS and PTSD for greater clarity in the construct.

Path analysis did not support the hypothesis that empathy, resilience and self-care contribute directly to STS in foster carers, but all three did contribute to CS (positively) and burnout (negatively). This lends credence to the theory of these predictors as coping resources contributing to the appraisal of stressors. Only self-care, however, showed a significant indirect effect on STS, which confirms the relevance of this factor in interventions with foster carers. Canfield’s (2005) theory that self-care should prevent the development of a chronic STS disorder is therefore applicable to foster carers as is Lazarus and Folkman’s (1984) link between coping strategies and health outcomes. Self-care also negatively predicted burnout, which suggests that it provides a counter-balance to burnout-engendered resource depletion (Kotaro et al. 2015). Self-care was further positively associated with CS, empathy and resilience, suggesting additional interactions between these variables. Further investigation of the contribution of different elements of self-care, perhaps in relation to different types of resource depletion, would help to expand the picture here. Eastwood and Ecklund (2008) found that the most relevant aspects of self-care for residential childcare workers were hobbies, reading and trips. While the present sample’s qualitative responses did mention all three aspects in relation to wellbeing maintenance, more frequently cited were time with others (particularly family), exercise, support and personal attitude. Time with others and support are consistent with findings that social support ameliorates indirect trauma (Michalopoulos and Aparicio 2012). The qualitative responses illustrate that what is understood to constitute self-care is likely to be highly personal, so caution should be exercised before applying generic self-care recommendations in training interventions.

Empathy scores did not directly predict STS, yet contributed positively to CS and negatively to burnout. While this would suggest an indirect effect on STS, this was not confirmed by multivariate analyses. For foster carers, empathy may have a more nuanced impact on overall professional quality of life via burnout and compassion satisfaction that should be further defined before empathy is recommended for inclusion in future interventions (see also Wagaman et al. 2015). The unidimensional TEQ measure used here included sympathetic physiological arousal, emotional comprehension in others and altruism, factors comparable to the dimensions of empathy which Wagaman et al. (2015) found predicted CS. Their study on social workers found that self-other awareness (cognitive) and affective response dimensions (related to mirror neurone mediated physiological response) predicted CS. Other dimensions of empathy which they found to predict STS (such as perspective taking) or burnout (emotion regulation) were not represented in the TEQ measure, and therefore empathy as a predictor of STS should not be discounted. For empathy to predict both CS and burnout in the present sample suggests a dual effect of contributing both risk and protection for professional quality of life in foster carers. Interventions for foster carers ought to include factors promoting CS as much as reducing risk of STS and burnout, again throwing doubt on empathy’s role in intervention design.

Resilience made the smallest contribution overall to CS (positively) and burnout (negatively). Neither direct nor indirect contributions to STS were supported. As with empathy, greater understanding of the impact of resilience is required before applying it to foster carer interventions which specifically target STS. Qualitative responses regarding maintenance of wellbeing from the sample may offer directions for further research. A prominent theme related to “personality or attitude” as a supportive resource may indicate deliberate employment of positive affective states, consistent with Ong et al. (2006). This has implications for training interventions, when considered alongside the recommendations of Hannah and Woolgar (2018): that foster carers receive training that promotes psychological flexibility. Resilience in the present study was measured as hardiness and persistence following adversity (Campbell-Sills and Stein 2007) and only one item specifically captured the employment of positive affective states. The present results do not support including resilience training in foster carer STS interventions, but do indicate the presence of a more complex underlying process.

Foster caring is a profession where arguably the boundaries between private and professional lives are particularly blurred. Still, while of increasing importance, little research has been devoted to this particular group among the caring professions. In our study, foster carers exhibited vulnerability to role-related STS, and burnout was central to its prediction. This affirms the importance of structural support and training to improve foster carer professional quality of life (Ottaway and Selwyn 2016). Foster carer exposure to primary trauma is also significant requiring relevant and sensitive support be made available to affected foster carers. Of the remaining predictors included, self-care is the most straight-forwardly applicable to foster carer interventions given its indirect effect on STS. While empathy and resilience contributed to CS and burnout, the pathways to STS were not clear, indicating the covariance between empathy, resilience and self-care being more complex.

### Limitations and Future Research Directions

Our findings come with several limitations, which need to be acknowledged. The present study was a non-randomised volunteer sample. It is possible that the stated survey aims (secondary trauma and burnout) may have influenced the type of respondent whereby foster carers experiencing higher levels of stress may have been more inclined to respond, and those who perceived STS as stigmatised may have opted not to respond. Other limitations are design-related. As a cross-sectional study, it is not possible to draw conclusions regarding the direction of effects represented by the correlations. Longitudinal studies would be beneficial to explore both the complex interaction between the three subscale measures of burnout, STS and CS, and the other predictor variables. Additionally, it would be beneficial to include the perspective of those who are being cared for. Of particular benefit would be to measure the extent of foster children’s exposure to trauma and the level to which foster carers engaged in therapeutic parenting, along with some of the effects on children. However, this is a hard-to-reach population, and the current findings in themselves advance substantially our understanding of predicting STS in this population.

In conclusion, our study evidenced the relevance particularly of burnout and self-care to foster carer interventions. The results lend support to STS preventative interventions which should (1) seek to reduce burnout through structural support and (2) encourage self-care to increase resources which may buffer the development of STS. While the direct contribution of empathy and resilience to STS variance was not supported, they both contributed positively, alongside self-care, to compassion satisfaction and burnout variance. Interventions for foster carers may be most effective, however, when they aim to both prevent STS and burnout, and at the same time promote CS.
